# Physiological and Growth Responses of Six Turfgrass Species Relative to Salinity Tolerance

**DOI:** 10.1100/2012/905468

**Published:** 2012-05-22

**Authors:** Md. Kamal Uddin, Abdul Shukor Juraimi, Mohd. Razi Ismail, Md. Alamgir Hossain, Radziah Othman, Anuar Abdul Rahim

**Affiliations:** ^1^Institute of Tropical Agriculture, Universiti Putra Malaysia, Selangor, 43400 Serdang, Malaysia; ^2^Department of Crop Science, Universiti Putra Malaysia, Selangor, 43400 Serdang, Malaysia; ^3^Department of Land Management, Universiti Putra Malaysia, 43400 Serdang, Malaysia

## Abstract

The demand for salinity-tolerant turfgrasses is increasing due to augmented use of effluent or low-quality water (sea water) for turf irrigation and the growing turfgrass industry in coastal areas. Experimental plants, grown in plastic pots filled with a mixture of river sand and KOSAS^R^ peat (9 : 1), were irrigated with sea water at different dilutions imparting salinity levels of 0, 8, 16, 24, 32, 40, or 48 dS m^−1^. Salinity tolerance was evaluated on the basis of leaf firing, shoot and root growth reduction, proline content, and relative water content. *Paspalum vaginatum* was found to be most salt tolerant followed by *Zoysia japonica* and *Zoysia matrella*, while *Digitaria didactyla*, *Cynodon dactylon* “Tifdwarf,” and *Cynodon dactylon* “Satiri” were moderately tolerant. The results indicate the importance of turfgrass varietal selection for saline environments.

## 1. Introduction

Salinity is a major abiotic environmental stress that is reported to be responsible for reducing plant growth across the globe. Sea water intrusion, in coastal states, has imposed salinity problems in turfgrass culture [1, 2]. Sodium chloride (NaCl) is the major compound contributing salinity in soils, and more salt-tolerant turfgrasses are required to cope this problem [3]. Therefore, development of salt-tolerant turfgrasses is becoming increasingly necessary in many parts of the world including Malaysia. Salt accumulation in soils, limitations on use of groundwater, and salt water intrusion into groundwater may restrict cultivation of glycophytic crops in these areas [4]. Salinity lowers water potential and restricts of water to plants [5]. Presence of excessive salt (NaCl) outside the cell can induce an osmotic stress, which may adversely affect the plant growth [6]. Hence, osmotic balance or osmoregulation is certainly a crucial factor for the survival of a plant under salt-stressed conditions. Generally, plants have developed different adaptive mechanisms to mitigate salinity under the saline environments [7–9]. Among these, salt exclusion is considered to be the most important adaptive feature of nonhalophytic plants, whilst most tolerant halophytes are salt accumulators [5]. Salt-accumulating halophytes are very crucial for osmotic adjustment. It could be achieved in the following ways: (i) by accumulating inorganic osmolyte (K^+^) and/or (ii) accumulating organic osmolytes such as proline. Therefore, salt-tolerant halophytic plants have the capability to minimize the detrimental effects by morphological means and physiological or biochemical processes [10].

Some of the turfgrass species are halophytic in nature. So salt-tolerant turf varieties would allow landscape development in saline environments and would be ideal in such environments, where limited or no fresh water is available for irrigation and salt water is the only option for irrigation practices. In addition, the use of sea water is also a good strategy for weed control in seashore *paspalum *worldwide. The native bermudagrass (*Cynodon dactylon*) here is quite salt-tolerant and grows vigorously, other salt-tolerant turfgrass species may also grow in the saline environments. In our previous reports [11, 12], several turfgrass species were identified in the coastal areas of Malaysia. Interestingly, the development of turfgrass industry especially in the coastal areas of Malaysia is an emerging field. To the best of our knowledge, published literatures are very scanty on salt tolerance studies in turfgrass species, which have been or being conducted in Malaysia. Therefore, this study was framed to determine the relative salinity tolerance and growth response of six important turfgrass species to salinity.

## 2. Materials and Methods

Glasshouse experiments were conducted at Faculty of Agriculture, University Putra Malaysia. Plastic pots (14 × 15 cm) were filled up with sandy soil (a mixture of river sand and peat; 9 : 1, v/v). The sandy soil had electrical conductivity (EC) 0.3 dS m^−1^, organic carbon 0.69%, sand 97.93%, silt 1.89%, and clay 0% with pH 5.23. The glasshouse temperature, relative humidity, and light intensity in morning time were 32°C, 80%, and 110 micromol m^−2^ s^−1^, and after noon 36°C, 70%, and 175 micromol m^−2^ s^−1^, respectively. The temperature was measured using a laboratory thermometer, and light intensity was monitored using a heavy duty light meter (Extech model 407026). Based on earlier findings of [13, 14], the three most salt-tolerant and three medium salt tolerant turfgrass species (Table 1) were used in this study.

The native soil was washed off the sods, and the sods were then transplanted into the plastic pots and grown for 8 weeks under nonsaline irrigation to achieve full growth. Three plants were transplanted in each pot. All species were narrow leaf and were clipped weekly at a cutting height of 5 mm. After 8 weeks thereafter, salinity treatments were initiated. Salinity treatments of 0, 8, 16, 24, 32, 40, and 48 dS m^−1^ (sea water) were applied. The control grasses were irrigated with distilled water. Sea water was diluted by adding distilled water to achieve different treatments. To avoid salinity shock, salinity levels were increased gradually by 8 dSm^−1^ day^−1^ for each treatment until the final salinity levels were achieved. After that, irrigation water was applied daily upto four weeks. The amount of water applied was 200 mL per pot. Data on leaf firing, proline, chlorophyll, relative water content, shoot and root dry weight were recorded 4 weeks after application of salinity treatment.

### 2.1. Determination of Leaf Firing

Leaf firing was estimated as total percentage of chlorotic leaf area, with 0% corresponding to no leaf firing and 100% for total brown leaves [15].

### 2.2. Determination of Shoot and Root Dry Weight

At the end of experiment (four weeks after salt initiation), shoots above the soil surface were harvested and washed with tap water and then distilled water to remove all soil particles. After harvesting the shoots, roots were removed from the soil, washed with tap water, and rinsed with distilled water. The shoot and root samples were then oven-dried to a constant weight at 70°C for 3 days. The dry weight (g/plant) was recorded for each treatment.

### 2.3. Determination of Proline Content

Proline was estimated following method of [16]. Fresh leaf tissue (0.5 g) was homogenized in 10 mL of 3% sulfosalicylic acid, and the homogenate was filtered through Whatman no. 2 filter paper. Two milliliters of the filtrate were brought to reaction with 2 mL acid ninhydrin solution (1.25 g ninhydrin in 30 mL glacial acetic acid), 20 mL orthophosphoric acid (6 M), and 2 mL of glacial acetic acid for 1 h at 100°C. The reaction was terminated in an ice bath. The reaction mixture was extracted with 4 mL toluene, mixed vigorously by passing a continuous stream of air for 1-2 min. The chromophore containing toluene was aspirated from the aqueous phase, warmed at room temperature, and the absorbance was recorded spectrophotometrically (Model UV-3101PC, UV-VIS NIR) at 520 nm. The proline concentration was determined from a standard curve and calculated on fresh weight basis as follows:

(1) $$\begin{array}{cc} & \mu \text{mol}\;\text{proline}\;{\text{g}}^{-1}\;\text{fresh}\;\text{weight}\\ & \;=\frac{\mu \text{g}\;\text{proline}\;{\text{mL}}^{-1}\;\times \;\text{mL}\;\text{of}\;\text{toluene}/115.5}{\text{g}\;\text{of}\;\text{sample}}. \end{array}$$

### 2.4. Determination of Chlorophyll Content

Chlorophyll content was estimated following method of [17]. Fresh leaves, from each pot, were cut into small pieces using a scissors and 200 mg of cut leaves were transferred into a plastic vial containing 20 mL of 80% acetone. The vial was quickly corked airtight and kept in the dark for 72 h. Absorbance of the solution was recorded at 645 and 663 nm spectrophotometrically (Model UV-3101PC, UV-VIS NIR). Chlorophyll content was estimated and expressed as mg g^−1^ of sample using the following formulae:

(2) $$\begin{array}{cc} & \text{Chlorophyll}\;\text{a}\;\text{content}\;\left(\text{mg}/\text{g}\;\text{fresh}\;\text{leaf}\right)\\ & \;\;=\frac{12.7\left(A_{663}\right)-2.69\left(A_{645}\right)}{1000}\times \frac{V}{W},\\ & \text{Chlorophyll}\;\text{b}\;\text{content}\;\left(\text{mg}/\text{g}\;\text{fresh}\;\text{leaf}\right)\\ & \;\;=\frac{22.9\left(A_{645}\right)-4.86\left(A_{663}\right)}{1000}\times \frac{V}{W},\\ & \text{Total}\;\text{chlorophyll}\;\text{content}\;\left(\text{mg}/\text{g}\;\text{fresh}\;\text{leaf}\right)\\ & \;\;=\frac{20.2\left(A_{645}\right)+8.02\left(A_{663}\right)}{1000}\times \frac{V}{W}, \end{array}$$

where *A*_645_ and *A*_663_ represent absorbance of solution at 645 and 663 nm, respectively, *V*: volume of the solution in mL, *W*: weight of fresh leaf sample in gram, 12.7, 2.69, 22.9, 4.86, 20.2, and 8.02 are absorption coefficients.

### 2.5. Determination of Relative Water Content

Relative water content (RWC) was determined as described by [18] on leaf tissues excised in the morning (around 9.00 am). Excised leaves from each pot (0.2 g) were measured for fresh weight (FW), and leaf samples were rehydrated in a water-filled petri dish for 4 h at room temperature. Turgor weight (TW) was measured by allowing full rehydration, removing all water from leaf surface, and weighing. Leaf dry weights were recorded after oven drying for one week at 60°C. The leaf relative water content was determined using the following formula:

(3) $$\text{RWC}=\frac{\text{Fresh}\;\text{weight}-\text{Dry}\;\text{weight}}{\text{Fully}\;\text{turgid}\;\text{weight}-\text{Dry}\;\text{weight}}\times 100.$$

### 2.6. Root Histology Using Scanning Electron Microscopy

Roots were sampled from two root zones (root tips at 0–50 mm from tip, and mature roots) and were cut into 5 mm portions with a sharp blade. The excised roots were placed in formalin acetic acid (FAA) and vacuumed for 1 h at 650 mm Hg. Specimens were postfixed in 1% osmium tetraoxide for 2 h, dehydrated for 30 min in each graded ethanol series at 30, 50, 70, 90, 95, and 100%, and dried in Baltec CPD 030 critical point dryer apparatus. The tissues were mounted on stubs, coated with gold using auto fine coater (JEOL JFC-1600, Japan) for 20 min, and viewed under a scanning electron microscope (JEOL JSM-5610LV, Japan), at high vacuum and acceleration voltage of 15 kV with a working distance of 23 mm.

### 2.7. Statistical Analysis

Data were analyzed statistically following randomized complete block design using ANOVA procedure in SAS statistical software (SAS). The treatment means were compared using protected least significant differences (LSD) at 5% level. Data of leaf firing was proportionate, so arcsine square root transformation was done.

## 3. Results

### 3.1. Leaf Firing

Interaction of salinity and species had a significant effect on leaf firing (Table 2). Leaf firing (%) increased with increasing salinity in all turfgrass species (Table 3). However, comparatively less salinity injury was recorded in* P. vaginatum, Z. japonica, *and* Z. matrella* compared to* D. didactyla, C. dactylon* “Tifdwarf,” and *C. dactylon* “Satiri” at all salinity levels. There was no injury (0%) recorded in all species up to 16 dS m^−1^ salinity, except for *D. didactyla* and* C. dactylon* “Tifdwarf” which showed light injury symptoms of 5 and 8%, respectively. At 24 dS m^−1^, the highest injury (25%) was recorded in* D. didactyla, *while the lowest injury of 5% was observed in* P. vaginatum. *At 32 dS m^−1^, leaf firing drastically increased to 79 and 75% in* D. didactyla *and* C. dactylon* “Tifdwarf,” respectively. At the highest salinity level of 48 dS m^−1^, the least leaf firing was observed in *P. vaginatum *(15%) followed by* Z. japonica *(25%) and* Z. matrella *(39%) compared to 80–100% leaf firing in* D. didactyla, C. dactylon* “Tifdwarf,” and *C. dactylon* “Satiri.” Overall, the highest leaf firing was recorded in *D. didactyla, *while the lowest in* P. vaginatum.*

### 3.2. Shoot Dry Weight

**Figure 1:**
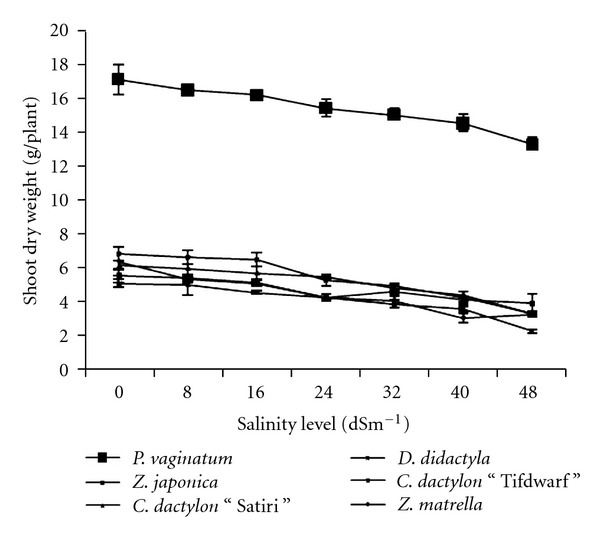
Shoot dry weight at different salinity levels of six turfgrass species.

Interaction effect of salinity and species was significant (*P* < 0.05) on shoot dry weight (Table 2). Shoot dry weights (SDWs) of turfgrass species decreased as the level of salinity increased (Figure 1). Results showed that* P. vaginatum* was the most salt-tolerant species being statistically significant with others. At the highest salinity level (48 dS m^−1^), SDW reduction in *P. vaginatum* was only 23% relative to control treatment. *Zoysia japonica *followed a similar trend as *P. vaginatum* for salinities upto 24 dS m^−1^. At 48 dS m^−1^, significantly higher SDW reductions were observed in *D. didactyla *(51%),* C. dactylon* “Tifdwarf” (53%), and *C. dactylon* “Satiri” (44%).

### 3.3. Root Dry Weight

**Figure 2:**
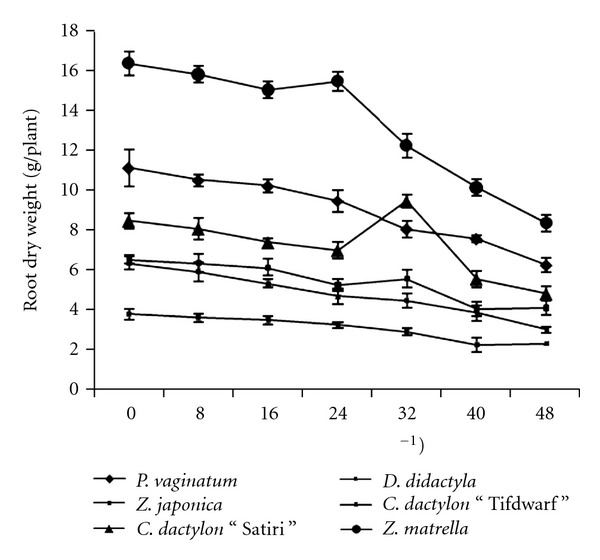
Root dry weight at different salinity levels of six turfgrass species.

The results showed that root dry weight (RDW) significantly (*P* < 0.05) decreased with increasing salinity (Figure 2). At 16 dS m^−1^, a significant difference was noted among the species. However, *P. vaginatum*, *C. dactylon* “Tifdwarf,” *Z. japonica,* and Z*. matrella* produced greater RDW than the others at 24 dS m^−1^ salinity. At the highest salinity (48 dS m^−1^), RDW reduction was least in *P. vaginatum *(34%) followed by* Z. japonica* (46%); while highest in *C. dactylon* “Tifdwarf” (67%) followed by* C. dactylon* “Satiri” (54%), Z*. matrella* (53%), and *D. didactyla* (47%). However, there were nonsignificant effect on root dry matter yield when salinity and species were interacted (Table 2).

### 3.4. Leaf Proline Content

Proline accumulation in the leaves of all turfgrass species increased with increasing salinity (Table 4). There were two distinct trends in proline accumulation among the species analyzed. In all turfgrass species (except* C. dactylon* “Satiri”), proline accumulation increased gradually up to 24 dS m^−1^ but increased abruptly at 32 and 48 dS m^−1^. At 48 dS m^−1^, a significantly higher (23.4-folds over the control) accumulation of proline was observed in *P. vaginatum *compared to in *C. dactylon* “Tifdwarf” (11.6-folds). There was a difference between the grasses with respect to proline accumulation patterns at 32 and 48 dS m^−1^. On the basis of proline accumulation ability, turfgrass species were ranked as *P. vaginatum > Z. matrella *>* D. didactyla *>* Z. japonica *> both of the* C. dactylon* entries. Interaction between salinity and species had also a significant (*P* < 0.001) effect on proline level (Table 7).

### 3.5. Leaf Relative Water Content (RWC)

Interaction effect of salinity and species was not significant for relative water content (Table 5). Relative water content (RWC) of all turfgrass species was significantly (*P* < 0.05) influenced by salinity. As salinity increased, RWC decreased. However, RWC for most of the species did not change up to 24 dS m^−1^ compared to the control (Table 5). Relative water content significantly decreased at 32 dS m^−1^ salinity level, except for* C. dactylon* “Satiri” and* Z. matrella*. According to reduction in RWC at 48 dS m^−1^ salinity level, species were ranked as* D. didactyla *(44.6%) >* C. dactylon* “Satiri” (42.7%) >* C. dactylon* “Tifdwarf” (37.5%) >* Z. matrella *(35.0%) >* Z. japonica *(33.7%) >* P. vaginatum *(21.3%).

### 3.6. Leaf Chlorophyll Content

Interaction effect of salinity and species was not significant for chlorophyll-a content (Table 7). Increasing salinity up to 24 dS m^−1^ did not affect chlorophyll-a content (Table 6). There were also no differences between 40 and 48 dS m^−1^ treatments on chlorophyll-b content, except for* D. didactyla*. In *P. vaginatum*, the chlorophyll-b content (0.11 mg g^−1^ FW) at 32 and 40 dS m^−1^ salinity levels was significantly different from other salinity levels (average 0.126 mg g^−1^ FW) (Table 7). In *Z. japonica, *a significant reduction in chlorophyll-b content was observed at 16 dS m^−1^, but there were no further reductions with increasing salinity.

Total chlorophyll content decreased under salt stress in different turfgrass species (Table 8). Interaction effect of salinity and species was significant (*P* < 0.05) for total chlorophyll (Table 7). Turf species with higher chlorophyll-a and chlorophyll-b contents, under control conditions, also had higher amounts of total chlorophyll. While *C. dactylon *“Satiri,” *C. dactylon *“Tifdwarf,” and *D. didactyla* had higher total chlorophyll under normal conditions, *P. vaginatum*, and *Z. japonica *maintained comparatively higher amounts of total chlorophyll under salt stress with marginal reductions compared to other turf species.

### 3.7. Root Cell Histology

**Figure 3:**
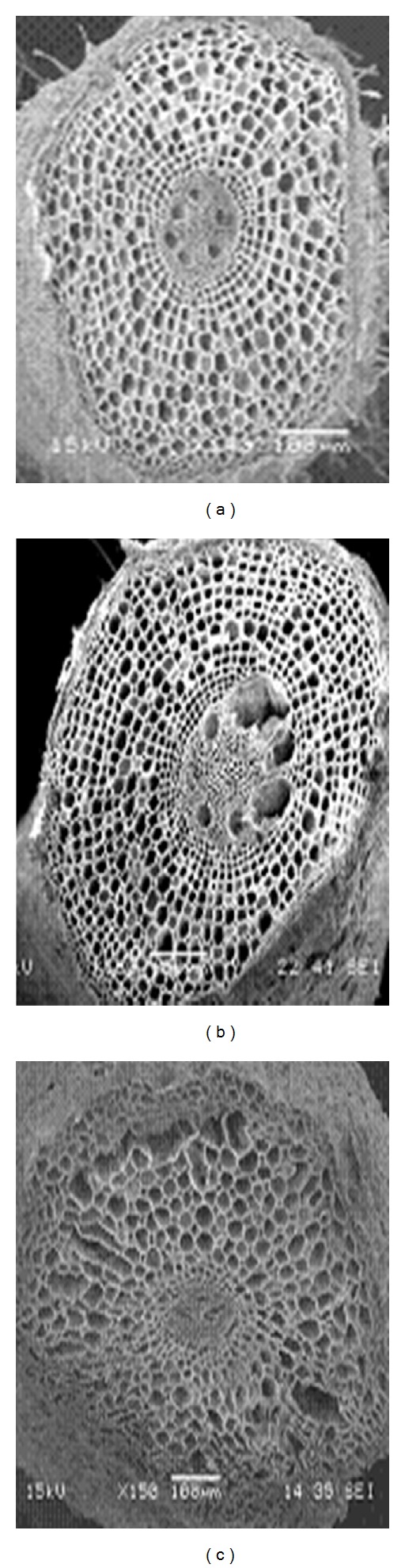
Scanning electron microscopy photographs showing root cortical tissue of *Paspalum vaginatum* under (a) 0, (b) 24, and (c) 48 dS m^−1^.

**Figure 4:**
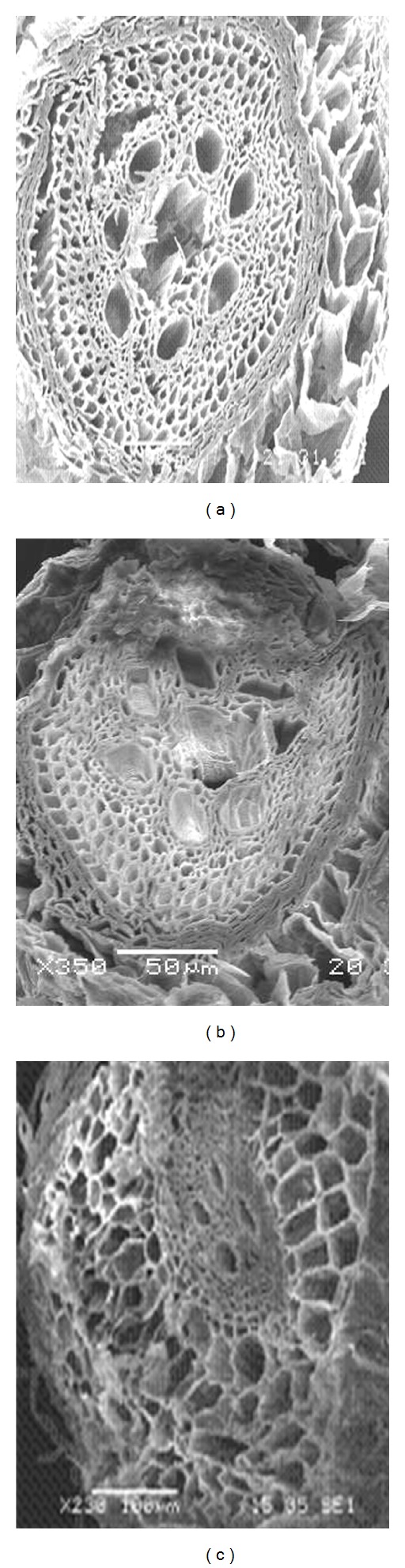
Scanning electron microscopy photographs showing root cortical tissue of *Zoysia japonica* under (a) 0, (b) 24, and (c) 48 dS m^−1^.

**Figure 5:**
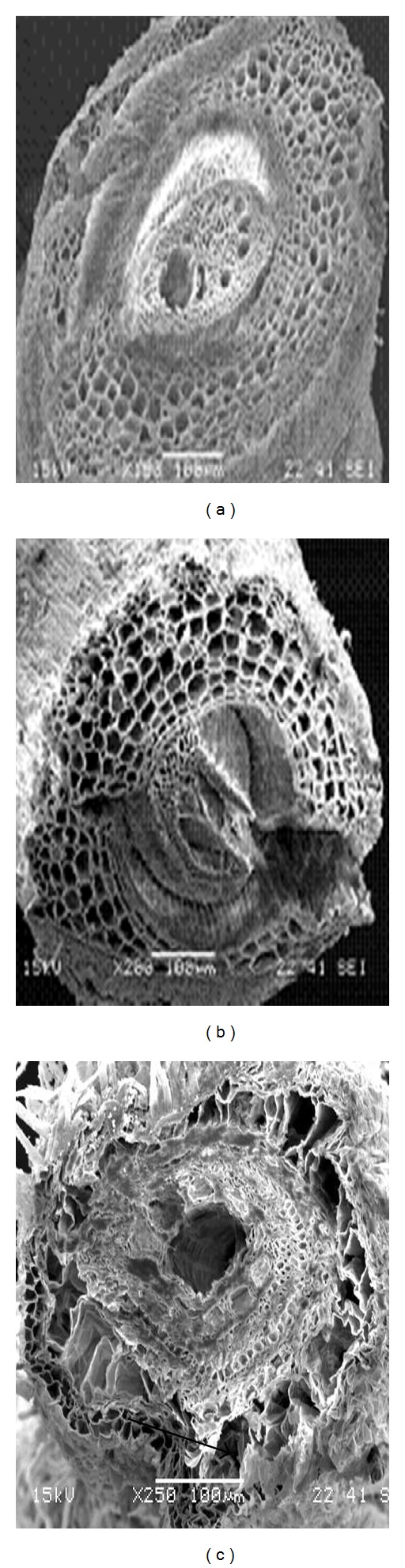
Scanning electron microscopy photographs showing root cortical tissue of *Zoysia matrella* under (a) 0, (b) 24, and (c) 48 dS m^−1^.

**Figure 6:**
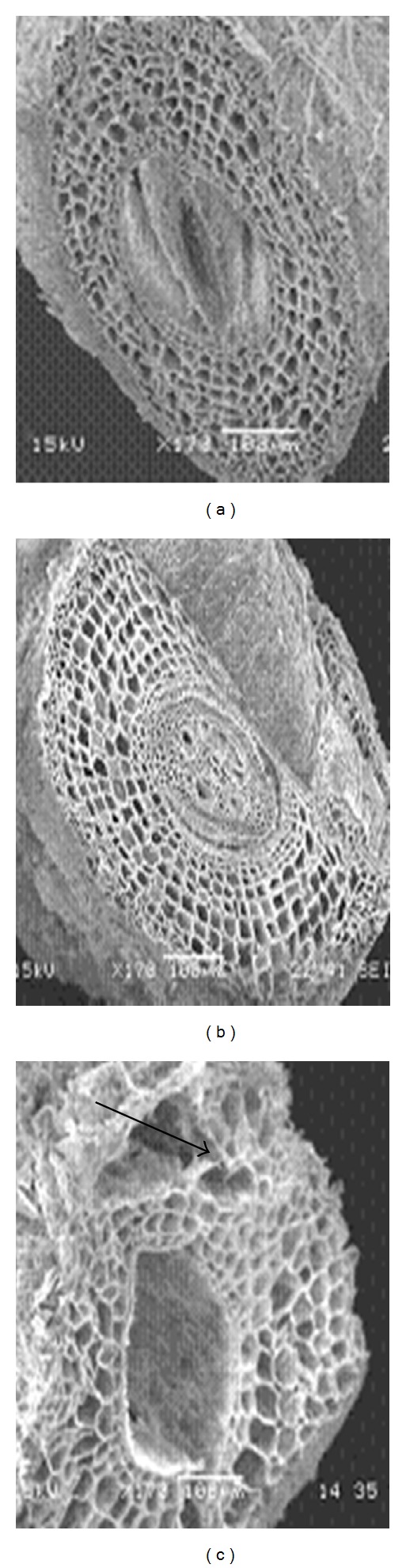
Scanning electron microscopy photographs showing root cortical tissue of *C. dactylon *“Tifdwarf” under (a) 0, (b) 24, and (c) 48 dS m^−1^. The arrow indicates cell damage (c) compared to control (a).

**Figure 7:**
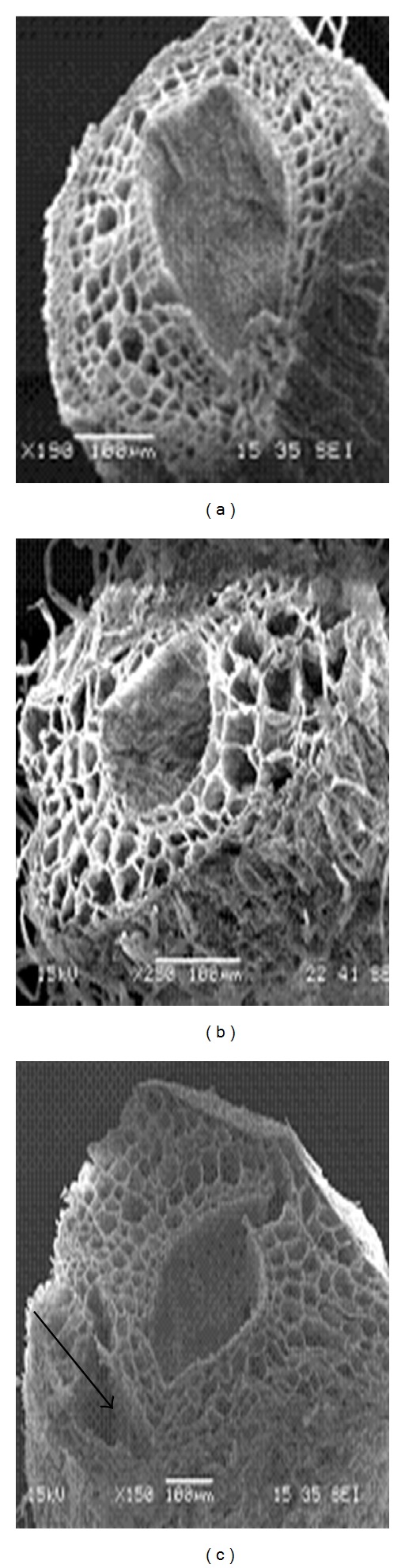
Scanning electron microscopy photographs showing root cortical tissue of *C. dactylon *“Satiri” under (a) 0, (b) 24, and (c) 48 dS m^−1^. The arrow indicates cell damage (c) compared to control (a).

**Figure 8:**
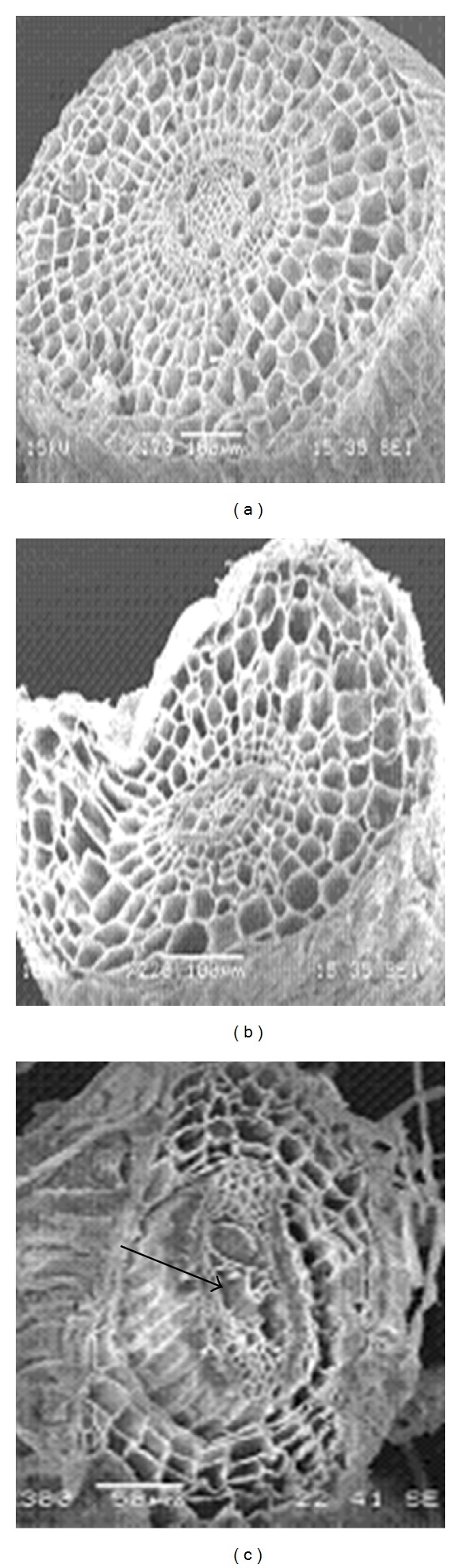
Scanning electron microscopy photographs showing root cortical tissue of *Digitaria didactyla* under (a) 0, (b) 24, and (c) 48 dS m^−1^. The arrow indicates cell damage (c) compared to control (a).

Differences in cell damage to root cortex of turfgrass species were observed. The damage resulted from cell collapse due to salt stress. Cortical cell of *P. vaginatum*, and *Z. japonica* did not show cell collapse in 24 and 48 dS m^−1^ salinity treatments (Figures 3 and 4).* Zoysia matrella* showed less cell collapse at 48 dS m^−1^ salinity treatment (Figure 5). *Digitaria didactyla, C. dactylon* “Tifdwarf,” and* C*.* dactylon *“Satiri” showed severe cell collapse at the highest salinity level (48 dS m^−1^) compared to the control (Figures 6, 7, and 8).

## 4. Discussion

The six turfgrass species in the present study exhibited a wide range in salinity tolerance in terms of dry matter production (Figures 1 and 2) and organic osmolyte accumulation (Table 4). In Malaysia, such type of research was not conducted ever before. Previously, we identified turfgrass species that were available in Malaysia and studied growth performance under salinity-stressed conditions [13, 14]. Throughout the globe, seashore paspalum exhibits a wide range of salinity tolerance among ecotypes [19–22]. A wide intraspecific variation in salinity tolerance has been reported to be as great as the interspecific variations [23]. Several researchers have reported that halophytes, which are ion includers, often adapt to low water potential by accumulation of inorganic solutes to maintain turgor pressure and total water potential [24–26].

 Salinity stressed plants certainly face osmotic challenges. This is in agreement with several previous reports [5, 19, 20, 27], which concur that osmotic adjustment is the main mechanism for survival and growth of plants under salinity stress. The percentage relative water content (RWC) was determined as an indicator of osmotic status of turfgrass species studied (Table 5). Halophytes are often able to accumulate high charges of salts in their tissues for osmotic adjustment through the compartmentalization of ions in vacuoles and the production of compatible solutes, or osmotic, in the cytoplasm [27]. Some compatible solutes that show an increase in concentration under salinity stress may also play significant role in osmotic adjustment, and these include proline, glycine betaine, and sugars [28–31]. Glycine betaine and proline protect enzymes (proteins) from damages caused by salinity or dehydration stress [32, 33]. Interestingly, significant proline accumulation generally occurs only after exceeding a threshold of drought or salt stress [30]. In the current study, salinity triggered proline synthesis in response to salinity to turgor maintenance (Table 4). Osmotic adjustment through synthesis of organic compounds has been postulated to have a significant role in salt tolerance in *P. vaginatum* [34]. Our studies indicated that salinity damaged root structure as a result of cortical cell collapse in *C. dactylon* “Tifdwarf,” *D. didactyla,* and *C. dactylon* “Satiri.” The structural damage in cortical tissue would interrupt radial water movement in the roots, thus limiting water uptake [35].

Chlorophyll degradation is the primary cause of photosynthetic degeneration/leaf firing and a main biochemical factor for the observed growth reduction [36]. The NaCl-induced decrease in chlorophyll level is widely reported in both glycophytes and halophytes [37–39]. In the present study, the chlorophyll damage was not recorded until 24 dS m^−1^ salinity level and thereafter chlorophyll damage increased with increasing salinity (Tables 6, 7, and 8). The chlorophyll degradation is associated with leaf firing (Table 3). Salinity-induced chlorophyll reduction may be related either to Mg deficiency and/or chlorophyll oxidation since reactive oxygen species (ROS) generation is common in salinity stressed conditions [40]. The chlorophyll-a content of all species decreased much more with increasing salinity (Table 6). However, [41] observed that salinity decreased chlorophyll-b content much more than chlorophyll-a. Chlorophyll content of *P. vaginatum* and *Z. japonica* seem to be insensitive to salinity up to 48 dS m^−1^. This is consistent with the earlier reports for other monocots including rice, wheat and maize chlorophyll-a by [42–44], chlorophyll-b and total chlorophyll contents decreased with increasing salinity [45], and salt-sensitive rice cultivars had lower chlorophyll content than salt-tolerant rice cultivars [45]. Similar observations were made by [46, 47].

## 5. Conclusion

The development of turfgrass industry in the coastal areas of Malaysia is challenging due to scarcity of fresh water for irrigation and salt tolerant weed species infestation. Sea water irrigation is a new technology widely used to suppress weed and maintaining the turfgrass growth simultaneously. Appropriate, realistic physiological criteria are essential to define the salinity tolerance and growth responses of turfgrass species. In the present study, salinity tolerance was evaluated on the basis of leaf firing, shoot and root growth reduction, proline content, and relative water content. We observed that *P. vaginatum* was highly salt tolerant at 48 dS m^−1^ followed by *Z. japonica* and *Z. matrella*, while *C. dactylon* “Tifdwarf” was least salt tolerant followed by *D. didactyla* and *C. dactylon* “Satiri.” The conclusions are based on responses of six turfgrass species to salinity. Many of the principles can be employed to discuss issues related to development of better direct selection criteria for other turfgrass species.

## Figures and Tables

**Table 1:** Turfgrass species used in this study.

Scientific name	Common name	Salt tolerance
*Paspalum vaginatum *Sw.	Seashore paspalum	Salt tolerant
*Zoysia japonica *Steud.	Japanese lawn grass	Salt tolerant
*Zoysia matrella *(L.) Merrill	Manila grass	Salt tolerant
*Cynodon dactylon *x. *Cynodon transvaalensis. *	Hybridbermuda grass (Satiri)	Medium salt tolerant
*Cynodon dactylon *x. *Cynodon transvaalensis. *	Hybridbermuda grass (Tifdwarf)	Medium salt tolerant
*Digitaria didactyla *Willd.	Serangoon grass	Medium salt tolerant

**Table 2:** Main effect and interaction effect on different variables by salinity and species.

Variable	Salinity	Species	Salinity × species
Leaf firing	1665.78***	513.16***	75.83***
Shoot dry weight	95.82***	1317.65***	4.01***
Root dry weight	79.83***	287.54***	1.15 ns
Proline	2176.10***	585.87***	58.07***
Relative water content	78.07***	13.85***	1.45 ns
Chlorophyll-a	30.03***	152.19***	0.89 ns
Chlorophyll-b	67.91***	78.03***	4.20***
Total chlorophyll	65.86***	206.75***	2.13***

Numbers are *F* values significant at ***P < 0.0001, ns: not significant.

**Table 3:** Effect of salinity on leaf firing of six turfgrass species.

EC_*w*_ (dS m^−1^)	Turfgrass species (% leaf firing)						
	*P. vaginatum*	*Z. japonica*	*Z. matrella*	*D. didactyla*	*C. dactylon *“Tifdwarf”	*C. dactylon *“Satiri”	LSD (0.05)
0	0 e (0.28)	0 e (0.28)	0 e (0.28)	0 f (0.28)	0 f (0.28)	0 e (0.28)	0.00
8	0 e (0.28)	0 e (0.28)	0 e (0.28)	0 f (0.28)	0 f (0.28)	0 e (0.28)	0.00
16	0 e (0.28)	0 e (0.28)	0 e (0.28)	5 e (12.79)	8 e (16.37)	0 e (0.28)	2.45
24	5 d (12.89)	10 d (18.26)	15 d (22.65)	25 d (29.90)	18 d (25.01)	15 d (22.65)	4.31
32	8 c (16.37)	15 c (22.65)	20 c (26.49)	79 c (63.17)	45 c (42.14)	25 c (29.95)	2.57
40	12 b (20.20)	20 b (26.52)	26 b (30.64)	93 b (76.80)	85 b (67.39)	69 b (56.37)	4.32
48	15 a (22.65)	25 a (29.95)	39 a (38.64)	100 a (89.75)	94 a (77.81)	80 a (63.83)	4.52

LSD (0.05)	2.31	2.34	2.30	6.19	4.36	5.11	

Means within columns followed by the same letter are not significantly different at *P* = 0.05 (LSD test).

Values in the parentheses indicate transformed by Arcsine square root.

**Table 4:** Effect of salinity on leaf proline content of six turfgrass species.

EC_*w*_ (dS m^−1^)	Turfgrass species (proline contents in mg g^−1^, fresh weight)						
	*P. vaginatum*	*Z. japonica*	*Z. matrella*	*D. didactyla*	*C. dactylon *“Tifdwarf”	*C. dactylon *“Satiri”	LSD (0.05)
0	3.33 f	3.60 d	3.67 f	3.55 e	5.60 f	6.35 e	0.96
8	4.60 ef (1.4)	4.07 d (1.1)	4.62 f (1.3)	6.42 e (1.8)	7.25 f (1.3)	10.60 e (1.7)	2.31
16	7.80 ed (2.3)	6.50 d (1.8)	6.02e (1.7)	12.40 d (3.5)	15.35 e (2.7)	29.90 d (4.7)	3.65
24	11.61 d (3.5)	13.10 c (3.6)	9.24 d (2.5)	15.05 d (4.2)	26.35 d (4.7)	52.50 c (8.3)	1.77
32	26.90 c (8.1)	16.25 c (4.5)	11.30 c (3.1)	34.55 c (9.7)	37.57 c (6.7)	66.52 b (10.5)	3.53
40	51.20 b (15.4)	45.82 b (12.7)	25.57 b (7.0)	43.27 b (12.2)	65.15 b (11.2)	71.35 a (11.2)	4.09
48	77.90 a (23.4)	49.62 a (13.8)	43.52 a (12.0)	49.92 a (14.1)	62.57 a (11.6)	74.85 a (11.8)	5.18

LSD (0.05)	4.45	3.26	1.26	3.49	1.93	4.43	

Means within columns followed by the same letter are not significantly different at *P* = 0.05 (LSD test).

Values in the parentheses indicate x-fold increase relative to the control.

**Table 5:** Effect of salinity on leaf relative water content of six turfgrass species.

EC_*w*_ (dS m^−1^)	Turfgrass species (relative water contents in %, fresh weight)						
	*P. vaginatum*	*Z. japonica*	*Z. matrella*	*D. didactyla*	*C. dactylon *“Tifdwarf”	*C. dactylon *“Satiri”	LSD (0.05)
0	93.16 a	89.48 a	89.89 a	87.33 a	90.85 a	90.18 a	8.83
8	90.24 ab	87.57 a	90.97 a	86.28 a	90.14 a	90.39 a	6.29
16	90.23 ba	85.22 a	86.87 a	84.78 a	85.02 ba	86.19 a	6.64
24	87.84 ba	84.92 a	88.51 a	82.42 a	83.91 ba	78.59 b	9.92
32	86.04 b bc	78.39 b	84.09 a	68.06 b	78.70 b	76.42 b	9.97
40	79.77 dc	72.28 c	73.28 b	63.46 b	65.27 c	64.85 b	9.51
48	78.68 b	66.30 d	64.98 c	55.35 c	62.51 c	57.30 c	8.03

LSD (0.05)	6.49	5.94	8.05	5.85	8.76	6.97	

Means within columns followed by the same letter are not significantly different at *P* = 0.05 (LSD test).

**Table 6:** Effect of salinity on chlorophyll-a concentration of six turfgrass species.

EC_*w*_ (dS m^−1^)	Turfgrass species (chlorophyll-a contents in mg g^−1^, fresh weight)						
	*P. vaginatum*	*Z. japonica*	*Z. matrella*	*D. didactyla*	*C. dactylon *“Tifdwarf”	*C. dactylon *“Satiri”	LSD (0.05)
0	0.49 a	0.40 a	0.36 a	0.30 a	0.49 a	0.57 a	0.070
8	0.47 a	0.39 ab	0.33 ab	0.29 a	0.48 a	0.57 a	0.059
16	0.46 ab	0.39 ab	0.31 abc	0.27 a	0.46 a	0.56 a	0.067
24	0.45 abc	0.38 ab	0.30 abc	0.26 a	0.45 ab	0.55 a	0.080
32	0.42 bc	0.35 bc	0.29 bc	0.20 b	0.40 bc	0.53 a	0.079
40	0.41 c	0.33 cd	0.26 c	0.19 b	0.35 cd	0.45 b	0.061
48	0.40 c	0.30 d	0.24 c	0.12 c	0.31 d	0.41 b	0.065

LSD (0.05)	0.051	0.042	0.066	0.063	0.052	0.077	

Means within columns followed by the same letter are not significantly different at *P* = 0.05 (LSD test).

**Table 7:** Effect of salinity on chlorophyll-b concentration of six turfgrass species.

EC_*w*_ (dS m^−1^)	Turfgrass species (chlorophyll-b contents in mg g^−1^, fresh weight)						
	*P. vaginatum*	*Z. japonica*	*Z. matrella*	*D. didactyla*	*C. dactylon *“Tifdwarf”	*C. dactylon *“Satiri”	LSD (0.05)
0	0.14 a	0.13 a	0.12 a	0.15 a	0.20 a	0.20 a	0.031
8	0.13 ab	0.13 a	0.11 b	0.13 ab	0.19 ab	0.19 ab	0.022
16	0.12 ab	0.10 b	0.10 b	0.13 ab	0.18 b	0.19 ab	0.017
24	0.12 ab	0.10 b	0.10 bc	0.12 ab	0.18 b	0.18 b	0.019
32	0.11 b	0.08 b	0.09 cd	0.10 bc	0.12 c	0.12 c	0.023
40	0.11 b	0.08 b	0.08 de	0.09 bc	0.09 d	0.11 cd	0.020
48	0.12 ab	0.08 b	0.06 e	0.08 c	0.11 cd	0.09 c	0.019

LSD (0.05)	0.024	0.026	0.014	0.033	0.018	0.018	

Means within columns followed by the same letter are not significantly different at *P* = 0.05 (LSD test).

**Table 8:** Effect of salinity on total chlorophyll concentration of six turfgrass species.

EC_*w*_ (dS m^−1^)	Turfgrass species (total chlorophyll contents in mg g^−1^, fresh weight)						
	*P. vaginatum*	*Z. japonica*	*Z. matrella*	*D. didactyla*	*C. dactylon *“Tifdwarf”	*C. dactylon *“Satiri”	LSD (0.05)
0	0.62 a	0.53 a	0.48 a	0.45 a	0.68 a	0.77 a	0.069
8	0.60 a	0.52 a	0.44 ab	0.42 ab	0.67 a	0.76 a	0.065
16	0.59 ab	0.49 a	0.41 b	0.40 ab	0.66 a	0.74 a	0.068
24	0.57 bac	0.48 ab	0.40 bc	0.38 b	0.63 ab	0.73 a	0.075
32	0.53 bc	0.41 bc	0.37 bc	0.29 c	0.52 bc	0.65 b	0.083
40	0.52 c	0.43 dc	0.34 cd	0.29 c	0.45 c	0.54 c	0.064
48	0.52 c	0.38 d	0.31 d	0.20 d	0.42 c	0.52 c	0.068

LSD (0.05)	0.060	0.050	0.079	0.068	0.100	0.074	

Means within columns followed by the same letter are not significantly different at *P* = 0.05 (LSD test).
